# Measuring health workers’ motivation in rural health facilities: baseline results from three study districts in Zambia

**DOI:** 10.1186/1478-4491-11-8

**Published:** 2013-02-21

**Authors:** Wilbroad Mutale, Helen Ayles, Virginia Bond, Margaret Tembo Mwanamwenge, Dina Balabanova

**Affiliations:** 1 Department of Community Medicine, University of Zambia School of Medicine, Keppel Street, London, WC1E 7HT, UK; 2 Clinical Research Department, Faculty of Infectious and Tropical Diseases, London School of Hygiene and Tropical Medicine, Keppel Street, London, WC1E 7HT, UK; 3 ZAMBART Project, Ridgeway Campus, University of Zambia, Nationalist Road, Lusaka, Zambia; 4 Department of Global Health and Development, Faculty of Public Health and Policy, London School of Hygiene and Tropical Medicine, 15-17 Tavistock Place, London, WC1H 9SH, UK

## Abstract

**Introduction:**

Health worker motivation can potentially affect the provision of health services. Low morale among the workforce can undermine the quality of service provision and drive workers away from the profession. While the presence of high-quality, motivated staff is a key aspect of health system performance, it is also one of the most difficult factors to measure.

**Methods:**

We assessed health worker motivation as part of the baseline assessment for a health system strengthening intervention in three rural districts in Zambia. The intervention (Better Health Outcomes Through Mentoring and Assessment (BHOMA)) aims to increase health worker motivation through training, mentoring and support. We assessed motivation by examining underlying issues grouped around relevant outcome constructs such as job satisfaction, general motivation, burnout, organization commitment, conscientiousness and timeliness that collectively measure overall levels of motivation. The tools and the concepts have been used in high-income countries and they were recently applied in African settings to measure health worker motivation.

**Results:**

Female participants had the highest motivation scores (female: mean 78.5 (SD 7.8) vs male: mean (SD 7.0)). By type of worker, nurses had the highest scores while environmental health technicians had the lowest score (77.4 (SD 7.8 vs 73.2 (SD 9.3)). Health workers who had been in post longer also had higher scores (>7 months). Health workers who had received some form of training in the preceding 12 months were more likely to have a higher score; this was also true for those older than 40 years when compared to those less than 40 years of age. The highest score values were noted in conscientiousness and timeliness, with all districts scoring above 80.

**Conclusions:**

This study evaluated motivation among rural health workers using a simple adapted tool to measure the concept of motivation. Results showed variation in motivation score by sex, type of health worker, training and time in post. Further research is needed to establish why these health worker attributes were associated with motivation and whether health system interventions targeting health workers, such as the current intervention, could influence health worker motivation.

## Introduction

Health worker motivation has the potential to affect the quality of health services. It has been recognized that low health worker morale can severely undermine demand for health services and may lead to wastage or loss of the limited number of workers [1,2]. In its 2006 World Health Report *Working Together for Health*, the World Health Organization (WHO) indicated a dramatic shift from understanding poor health worker performance as being caused by lack of knowledge and skills to a focus on health workers’ motivation and on management of the workforce [3,4]. The report emphasized the need to develop capable, motivated and supported health workers. This is an essential ingredient in overcoming bottlenecks to achieving national and global health goals [3,4]. In recent years there has been an upsurge of interest in human resources required to deliver healthcare in low-income settings in an effort to achieve targets for the UN Millennium Development Goals [5]. Much of the attention has been on the inadequate numbers of healthcare workers and their inequitable distribution [4-9]. However, it is increasingly appreciated that attention must also be paid to health worker performance and motivation [10-12].

Although it has been accepted that the presence of high-quality and motivated staff is essential for provision of quality healthcare, at the same time it has also been acknowledged that this is one of the most difficult inputs to assess and ensure [11]. Health worker job satisfaction, which can be defined as ‘the attitude towards one’s work and the related emotions, beliefs, and behaviors’, results from complex interactions between on-the-job experience, organizational environment and motivation [13]. Motivation is defined as an individual's degree of willingness to exert and maintain an effort towards attaining organizational goals [13]. Job satisfaction is inextricably linked to motivation and both involve cognitive, affective, and behavioral processes, with worker motivation commonly understood as the reason why workers behave as they do towards achieving personal and organizational goals. Neither job satisfaction nor motivation is directly observable, but both have been identified as critical to the retention and performance of health workers [12,13].

Many factors that range from available physical infrastructure to an individual's highly personal values are known to influence the performance of health professionals [11,14]. It is likely that motivation influences performance directly and mediates or modifies the effect of interventions aimed at changing performance; however, there are few studies on its influence on practice change in health workers in low-income settings [11,14]. The existing studies have focused predominantly on determinants of motivation, with less of the literature focusing on conceptualizing and measuring worker motivation.

Some authors have contended that the main determinant of health sector performance is health worker motivation, and while resource availability and worker competence are necessary, they are not sufficient [14]. In addition to technical training, health workers must work in environments with incentives in place that reward high-quality performance. To this end, an understanding of employee motivation is necessary to design systems with the right incentives [15].

In Zambia, a study performed in the context of HIV services in urban health facilities within the public sector showed that 50% of health workers met the definition of being in burnout and many health workers complained of poor pay, stress and work overload. Most of them confirmed that they were prone to leaving the current post in search of greener pastures in non-governmental organizations (NGOs) and the private sector [16].

Within the Zambian government system, there are 9 Provincial Health Offices, 72 District Health Offices, 98 hospitals, 265 urban health centers, 1,029 rural health centers, and 171 health posts. Health centers are intended to serve 30,000 to 50,000 people in urban areas and 10,000 people in rural areas, with a 29-km radius catchment area. Human resource challenges for the health sector in Zambia are well documented [17]. Shortage of skilled health workers constitutes a very important bottleneck to service delivery. According to records from the Ministry of Health (MOH), the total number of staff in the health sector stands at 29,533, which is 57 percent of the approved establishment. Less than 50% of frontline health workers (nurses, midwives, clinical officers, environmental health technicians (EHTs)) are available in relation to need to provide primary healthcare [18].

Public facilities in rural and remote areas have the lowest number of health workers compared to urban areas [18]. The result is that there are a number of Health Posts and Rural Health Centers in rural and remote areas that are run by unqualified staff or have only one qualified staff member [17,18].

In this study we adopted a 23-item score to measure health workers motivation as part of the baseline for a health-system-strengthening intervention in 3 rural districts of Zambia. Our aim was to determine the applicability of the motivation tool in the Zambian healthcare context, especially among rural health workers in rural health facilities, with a view to using the tool in subsequent monitoring of change in motivation after the implementation of health system interventions described elsewhere (Mutale *et al*., unpublished, [19]. The tool used and the underlying theoretical concepts have been used in high-income countries [13,20,21] and have recently been adapted and used in Kenya among hospital health workers [22]. However, this tool has not been applied in small rural heath facilities where motivation determinants may be different from those working in hospitals.

## Methods

This work is part of a larger study in Zambia known as Better Health Outcomes through Mentoring and Assessment (BHOMA), which is a stepped wedge community randomised controlled trial that aims to strengthen the health system in three rural districts of Zambia. The BHOMA intervention is being implemented in Chongwe, Luangwa and Kafue Districts, all in Lusaka Province, Zambia. The combined population for the 3 districts is 306,000, with a total of 48 health facilities and 4 general hospitals. Two separate but complementary packages are being applied in the BHOMA intervention: the health facility package (which targets the health workers and their support staff through training, mentoring and support) and the community-based package (which works within the community to improve access to health services and improve data and referral systems).

The BHOMA intervention is complex and labor intensive, and is therefore being rolled out gradually from one health facility to the next over a period of 3 years using a stepped wedged design [23,24]. The full intervention and the evaluation design are described elsewhere (Mutale *et al*., unpublished [19]. A baseline health facility survey was conducted in 42 out 48 health facilities found in the 3 BHOMA districts between January and April 2011. This constituted 96% of the total health facilities, with the rest being used as pilot sites for the BHOMA intervention.

In this study, we interviewed 1 to 3 health workers at each of the 42 health facilities who were present at the time of baseline data collection, depending on the available staff. Most health facilities had just one eligible health worker. Where there were more than three, up to three health workers were randomly selected to take part in the study. They were eligible if they had been working in the facility for at least 1 month and were attending to patients. All participants were given instructions about the tool, which was self-administered though the respondents were free to clarify questions that they did not understand. Before being used in the Zambian setting, the tool was pretested and questions were adapted to suit the lower level health facilities but the content remained essentially the same as described by Mbindyo *et al*. [22].

The data collection tool was selected as it was easy to use and there is no available tool that has been used in Zambia previously. It is hoped that the assessment will be repeated after 12 months in the same health facilities to determine any changes. The tool had 23 items, with answers given on a scale of 1 to 5 (strongly agree to strongly disagree) (Table 1). The items with negative statements were reverse coded when calculating scores.

**Table 1:** **Mean scores for the 23**-**item motivation construct**

**Category**	**Description**	**Mean score (1 to 5)**
General motivation	Feel motivated to work hard	2.97
	Only do this job to get paid	3.95
	I do this job as it provides long-term security for me	2.99
Burnout	I feel emotionally drained at the end of the day	3.02
	Sometimes when I get up in the morning, I dread having to face another day at work	3.46
Job satisfaction	Overall, I am very satisfied with my job	3.71
	I am not satisfied with my colleagues in my work	3.74
	I am satisfied with my supervisor	3.92
Intrinsic job satisfaction	I am satisfied with the opportunity to use my abilities in this job	4.00
	I am satisfied that I accomplish something worthwhile in this job	4.17
	I do not think that my work in this health facility is valuable these days	3.95
Organization commitment	I am proud to be working for this health facility	4.02
	I find that my values and this health facility are very similar	3.60
	I am glad that I work for this facility rather than other facilities in the country	3.05
	I feel very little commitment to this health facility	3.98
	This health facility really inspires me to do my very best on the job	3.52
Conscientiousness	I cannot be relied on by my colleagues at work	4.34
	I always complete my tasks efficiently and correctly	4.08
	I am a hard worker	4.59
	Do things that need doing without being asked or told	4.44
Timeliness	I am punctual about coming to work	3.98
	I am often absent form work	4.58
	It is not a problem if I sometimes come late for work	4.09

The scale for negatively worded questions was reverse coded so that 1 was ‘strongly agree’ and 5 ‘strongly disagree’. Thus, a high score shows disagreement with a negative statement and is therefore suggestive of higher motivation.

Data was entered into a Microsoft access database (Microsoft, Redmond, WA, USA) and exported to SPSS version 19 (SPSS, Chicago, IL, USA) for analysis. Factor analysis was used to confirm latent factors described by Mbindyo *et al*. [22]. The scores were standardized to 100 in order to allow for comparison between subscores. The overall scores were calculated by the sum of all subscores of the latent factors described. Linear regression was used to identify determinants of motivation.

### Ethical considerations

The study was approved by the University of Zambia Bioethics Committee and the London School of Hygiene and Tropical Medicine Ethics Committee. All respondents were informed about the purpose of the survey and were asked to sign a consent form before taking part in the study. Confidentiality was ensured during data collection and subsequent publication of the results.

## Results

In total, 96 health workers completed the self-assessment tool and none of the health eligible health workers refused to participate, giving a 100% response rate. Most of the participants were from Chongwe district, a reflection of the number of health facilities in that district compared to the other two districts. Luangwa had the lowest number of participants (13 (13.5%)) as it had fewer health facilities. In terms of sex distribution, there were more female respondents (41/96 (58%)) compared to males (42%). The majority of the health workers were between 30 to 40 years of age (29/96 (30%)). The skill mix included nurses who were twice as numerous as clinical officers (38/96 (38%) versus 18/96 (18%), respectively). Untrained workers who nonetheless attended to patients (classified daily employees) made up 11/96 (12%). The majority of the respondents had been in post for more than 12 months. A third of the respondents (30/96 (31%)) reported never having attended any training in the preceding 12 months (Table 2).

**Table 2:** Demographic characteristics of the health workers recruited in the motivation evaluation

**Variable**	**n**	**%**
District:		
Chongwe	54	56.3
Kafue	29	30.2
Luangwa	13	13.5
Sex:		
Male	41	42.7
Female	55	57.3
Age group:		
20 to 29	25	26.0
30 to 39	29	30.2
40 to 49	18	18.8
≥50	24	25.0
Role:		
Nurse	36	37.5
Clinical officer	18	18.8
Environmental health technician	16	16.7
Classified daily employee	11	11.5
Other workers	15	15.6
Time in post:		
3 months	6	6.3
4 to 6 months	1	1.0
7 to 12 months	14	14.6
More than 12 months	75	78.1
Received training past 12 Months		
No	30	31.3
Yes	66	68.8

The 23 items as an index of motivation had a Cronbach’s α of 0.73. The highest scores were for item 19 (being a hard worker) and disagreement with the statement of being absent from work (item 22) (Table 1).

Female participants had the highest motivation scores (female mean 78.5 (SD 7.8) vs male mean 74.1 (SD 7.0)) By role, nurses had the highest scores while EHTs had the lowest mean score (nurses 77.4 (SD 7.8) vs EHT 73.2 (SD 9.3)).

Those who had received some form of training in the preceding 12 months were more likely to have a higher motivation score. This was true for those older than 40 years when compared to those less than 40 years of age (Table 3).

**Table 3:** Overall motivation scores stratified by demographic characteristics

**Variable**	**N**	**Overall mean score**	**SD**
District:			
Chongwe	54	88.76	8.87
Kafue	29	85.97	9.57
Luangwa	23	90.54	7.47
Residence:			
Peri-urban	20	86.70	8.55
Rural	70	88.61	9.39
Hospital	6	87.67	4.76
Sex:			
Male	41	74.10	7.04
Female	55	78.56	7.85
Role:			
Nurse	36	77.44	7.82
Clinical officer	18	74.78	6.36
Environmental health technician	16	73.15	9.31
Classified daily employee	11	76.99	6.97
Other workers	15	80.52	6.86
Time in post:			
3 months	6	74.78	13.46
4 to 6 months	1	70.43	0.00
7 to 12 months	14	75.90	7.37
More than 12 months	75	77.03	7.43
Received training past 12 Months			
Yes	66	77.59	7.15
No	30	74.61	8.85
Age group:			
20 to 29	25	75.79	9.43
30 to 39	29	74.15	7.38
40 to 49	18	78.84	6.38
≥50	24	78.95	6.65

Generally, female participants had the highest scores across all subcategories of motivation latent factors except for timeliness, which showed a mixed picture. The highest scores were noted for conscientiousness and timeliness, with all districts scoring above 80%. The lowest scores were for burnout, all below 70. Females in Luangwa and Kafue scored fairly highly in most categories. When comparisons were made among male participants, Luangwa had the highest scores across six of the seven categories. This was followed by Chongwe district (Table 4).

**Table 4:** Mean standardized motivation subscores by latent factors stratified by district and gender

**Category**	**Chongwe (n = 54)**		**Kafue (n = 29)**		**Luangwa (n = 13)**	
	**Male (n** **=** **22)**	**Female (n** **=** **32)**	**Male (n** **=** **14)**	**Female (n** **=** **15)**	**Male (n** **=** **5)**	**Female (n** **=** **8)**
General motivation	63.94	66.66	63.81	70.22	52.00	74.17
Burnout	63.64	67.19	62.14	64.00	68.00	62.50
Job satisfaction	75.45	79.17	70.48	71.56	72.00	82.50
Intrinsic job satisfaction	78.48	82.29	74.76	82.67	85.33	85.00
Organization commitment	66.73	79.12	64.86	73.87	72.80	75.00
Contentiousness	85.00	88.59	86.43	86.67	96.00	85.63
Timeliness	86.36	82.91	80.95	84.44	88.00	88.33

In all, 21 items had a coefficient value of more than 0.4, which was used as a cut off point for further analysis. This cut-off means that each item has a shared variance of at least 16% with the factor under consideration [25]. Using these criteria, seven latent factors were confirmed from factor analysis. The highest loading was for the timeliness latent factor. Intrinsic job satisfaction and organization commitment and general motivation factors also loaded highly on the factor analysis. Two items loaded less that 0.4, and this is shown by dashes in Table 5.

**Table 5:** Factor analysis of health worker motivation

**No**.	**Description**	**General motivation**	**Burnout**	**Job satisfaction**	**Intrinsic job satisfaction**	**Organization commitment**	**Contentiousness**	**Timeliness**
1	I Feel motivated to work hard	0.563						
2	I Only do this job to get paid	0.623						
3	I do this job as it provides long-term security for me	0.719						
4	I feel emotionally drained at the end of the every day		−0.789					
5	Sometimes when I get up in the morning, I dread having to face another day at work		0.540					
6	Overall, I am very satisfied with my job			0.721				
7	I am not satisfied with my colleagues in my work			-				
8	I am satisfied with my supervisor				0.790			
9	I am satisfied with the opportunity to use my abilities in this job				0.678			
10	I am satisfied that I accomplish something worthwhile in this job				0.569			
11	I do not think that my work in this health facility is valuable these days				0.697			
12	I am proud to be working for this health facility					0.717		
13	I find that my values and this health facility are very similar					0.718		
14	I am glad that I work for this facility rather than other facilities					0.633		
15	I feel very little commitment to this health facility					0.601		
16	This health facility really inspires me to do my very best on the job					0.626		
17	I cannot be relied on by my colleagues at work						0.649	
18	I always complete my tasks efficiently and correctly						-	
19	I am a hard worker						0.727	
20	Do things that need doing without being asked or told						0.715	
21	I am punctual about coming to work							0.824
22	I am often absent from work							0.776
23	It is not a problem if I sometimes come late for work							0.838

Extraction method was principal component analysis. Rotation method was varimax with Kaiser normalization.

The linear regression model revealed that the major determinants of higher motivation were female gender (coefficient: 5.8, *P* = 0.008) and working in non-clinical areas (for example, pharmacists or laboratory technicians, coefficient: 6.9, *P* = 0.039). Univariate analysis showed that age and belonging to a hospital-based health facility were associated with higher motivation scores, but these were not statistically significant in the full model (Table 6).

**Table 6:** **Linear regression model for the predicators of health worker motivation score** (**N** = **96**)

**Predictor**	**n**	**Coefficient**	**SE**	***P *value**
Constant		73.199	4.87	0.000
Time in post:				
Less than 6 months	7	-		
7 to 12 months	14	−3.004	4.34	0.491
More than 12 months	75	−0.798	3.60	0.825
District:				
Kafue	54	-		
Chongwe	29	1.459	2.11	0.491
Luangwa	23	4.095	3.01	0.178
Residence:				
Peri-urban	20	-		
Rural	70	3.171	2.40	0.192
Hospital*	6	−0.681	4.44	0.878
Received training?				
No	30	-		
Yes	66	2.896	2.09	0.170
Sex:				
Male	41			
Female	55	5.778	2.12	0.008
Type of health worker:				
Environmental health technician	36	-		
Nurse	18	0.341	2.97	0.909
Clinical officer	16	3.445	3.11	0.271
Classified daily employee	11	1.156	3.49	0.741
Non-clinical	15	6.909	3.29	0.039
Age	96	0.133	0.086	0.127

Overall *P* = 0.036, R^2^ = 0.236.

## Discussion

Motivation of health workers is key to providing good quality and accessible healthcare and achieving UN Millennium Development Goals, especially in rural communities where most of the indicators are lagging behind [18]. The results of this study could be useful, especially in the Zambian context where healthcare human resource challenges continue to hamper provision of quality services [18]. Our study has demonstrated that it is feasible to measure motivation among health workers working in very deprived and rural communities in Zambia using a simple adapted tool. It was important to validate the tool in the local context especially as it has never been used in Zambia to measure motivation among health workers. Our results also indicate that the tool could be made even simpler, as suggested by Mbindyo *et al*., from 23 items to about 10 to 12 items based on item loadings on factor analysis [22]. Our experience with the tool was that it was easy to use and most health workers did not have problems answering the questions. However, we noted that there was a tendency towards preference for higher scores, hence affecting the mean scores which were generally on the higher side with overall and subscores all above 60. This could be attributed to response bias, where the respondents tended to give higher rates as they felt this was desired [22,26].

The overall motivation patterns showed interesting variations that will be further explored when comparing the intervention and control health facilities during follow-up studies. The baseline results showed that mean motivation scores varied by sex, type of health worker, training and district. Time in post and age also showed variation in motivation scores. Further studies are required to establish why these attributes were important in explaining health worker motivation.

In terms of sex variation, motivation scores for females tended to be higher than that of male participants. Regression analysis showed significant association between motivation and female gender. Similar results have been reported in Ethiopia, where female health workers were more likely to report work satisfaction compared to males [27]. However, it is possible to speculate in terms of what motivates different genders in general. It has been recognized that men are more motivated by higher wages and prestigious jobs while women are more concerned with job security and community value for the work they do [28]. The rural environment and the poor working conditions in the health sector in Zambia seemed to have less effect on women compared to men.

Among the health workers, nurses were highly motivated when compared to clinical officers and environmental health technicians. This could be attributed to the higher number of women among nurses and the higher number of men among the less motivated groups of clinical officers and environmental health technicians. Interestingly, untrained health workers attending to patients, known as classified daily employees (CDEs), appeared to be more motivated when compared to clinical officers and environmental health technicians. This could be attributed to the fact that the CDEs may have less expectation and have other things on which they based their motivation, including appreciation by the community. More research is need to establish why CDEs appeared more motivated and whether such motivation is sustainable especially at a time when task shifting and use of lay community workers is being advocated [29-31].

The finding that non-clinical health workers (such as pharmacists and environmental health technicians) had significantly higher motivation scores agrees with the Kenyan motivation study where they also noted higher motivation among non-clinical health workers [22]. This could be related to workload, which is usually more for clinician workers and could negatively affect their motivation [32,33].

Another observation was that the longer the heath workers stayed in post the more motivated they were. This was also true for age, where older health workers had higher motivation scores than younger ones [28]. It appeared that those who had stayed longer had settled and integrated well within their community, while newcomers were faced with the challenges of working and settling in rural settings after completing training in urban training schools. This finding is crucial when discussing health worker retention schemes. The focus might be to ensure retention and reduce turnover, which is associated with many newcomers and fewer staff staying longer and hence missing out on the stability and motivation that is associated with a longer stay and age maturity [34].

One other critical finding was that those who had attended some form of training in the preceding 12 months were more likely to have higher scores when compared to those who had never attended any training. Literature has shown that in-service training could be a motivating factor for health workers rather than just a focus on higher wages. This study seems to support the need for continuous but systematic refresher training as a source of both skills and motivation [35,36]. It will be interesting to establish whether motivation scores change with the training and mentoring intervention targeting health workers in the BHOMA trial. This will be the next stage of our ongoing work.

The limitations of our study include that it does not link motivation to service delivery in order to establish any possible causal link. This was not within the scope of the current paper. Another limitation was that we used subjective methodology to collect data from health workers and it was possible that respondents could have been tempted to give high scores, thus biasing the results. It must also be noted that motivation was measured among only 96 health workers. It is recommended to repeat the study with a larger sample size.

## Conclusions

This study evaluated motivation levels among rural health workers using a simple adapted tool to measure the concept of motivation. The results showed variation in motivation score by gender, type of health worker, training and time in post. Further research is needed to establish why these health worker attributes were associated with motivation and whether health system interventions such as the current BHOMA initiative, can influence health worker motivation in the short or long term.

## Competing interests

The authors declare they have no competing interests.

## Authors’ contributions

WM: conceived the paper, analyzed the data and wrote the initial draft. HA: conceived the project and reviewed the draft and final manuscript. GB: provided critical analysis and contributed to the writing of the paper. MTM: contributed to writing of the paper and provided materials for the evaluation design. DB: involved in the draft of the paper and provided critical analysis of the scientific content. All authors read and approved the final manuscript.
